# Ageing contributes to phenotype transition in a mouse model of periodic paralysis

**DOI:** 10.1002/rco2.41

**Published:** 2021-05-05

**Authors:** Karen J. Suetterlin, S. Veronica Tan, Roope Mannikko, Rahul Phadke, Michael Orford, Simon Eaton, Avan A. Sayer, Miranda D. Grounds, Emma Matthews, Linda Greensmith, Michael G. Hanna

**Affiliations:** ^1^ Department of Neuromuscular Diseases UCL Queen Square Institute of Neurology London UK; ^2^ MRC Centre for Neuromuscular Diseases UCL Queen Square Institute of Neurology and National Hospital for Neurology and Neurosurgery London UK; ^3^ AGE Research Group, NIHR Newcastle Biomedical Research Centre Newcastle upon Tyne Hospitals NHS Foundation Trust and Newcastle University Newcastle upon Tyne UK; ^4^ Guy's and St Thomas' NHS Foundation Trust London UK; ^5^ Department of Neuropathology Great Ormond Street Hospital London UK; ^6^ UCL Great Ormond Street Institute of Child Health London UK; ^7^ School of Human Sciences University of Western Australia Perth Australia; ^8^ Atkinson Morley Neuromuscular Centre, Department of Neurology St Georges University Hospitals NHS Foundation Trust London UK

**Keywords:** Ageing; Channelopathy; Periodic paralysis; Sarcopenia; Skeletal muscle; Ion channels

## Abstract

**Background:**

Periodic paralysis (PP) is a rare genetic disorder in which ion channel mutation causes episodic paralysis in association with hyper‐ or hypokalaemia. An unexplained but consistent feature of PP is that a phenotype transition occurs around the age of 40, in which the severity of potassium‐induced muscle weakness declines but onset of fixed, progressive weakness is reported. This phenotype transition coincides with the age at which muscle mass and optimal motor function start to decline in healthy individuals. We sought to determine if the phenotype transition in PP is linked to the normal ageing phenotype transition and to explore the mechanisms involved.

**Methods:**

A mouse model of hyperkalaemic PP was compared with wild‐type littermates across a range of ages (13–104 weeks). Only male mice were used as penetrance is incomplete in females. We adapted the muscle velocity recovery cycle technique from humans to examine murine muscle excitability *in vivo*. We then examined changes in potassium‐induced weakness or caffeine contracture force with age using *ex vivo* muscle tension testing. Muscles were further characterized by either Western blot, histology or energy charge measurement. For normally distributed data, a student's *t*‐test (± Welch correction) or one‐ or two‐way analysis of variance (ANOVA) was performed to determine significance. For data that were not normally distributed, Welch rank test, Mann Whitney U test or Kruskal–Wallis ANOVA was performed. When an ANOVA was significant (*P* < 0.05), post hoc Tukey testing was used.

**Results:**

Both WT (*P* = 0.009) and PP (*P* = 0.007) muscles exhibit increased resistance to potassium‐induced weakness with age. Our data suggest that healthy‐old muscle develops mechanisms to maintain force *despite* sarcolemmal depolarization and sodium channel inactivation. In contrast, reduced caffeine contracture force (*P* = 0.00005), skeletal muscle energy charge (*P* = 0.004) and structural core pathology (*P* = 0.005) were specific to Draggen muscle, indicating that they are caused, or at least accelerated by, chronic genetic ion channel dysfunction.

**Conclusions:**

The phenotype transition with age is replicated in a mouse model of PP. Intrinsic muscle ageing protects against potassium‐induced weakness in HyperPP mice. However, it also appears to accelerate impairment of sarcoplasmic reticulum calcium release, mitochondrial impairment and the development of core‐like regions, suggesting acquired RyR1 dysfunction as the potential aetiology. This work provides a first description of mechanisms involved in phenotype transition with age in PP. It also demonstrates how studying phenotype transition with age in monogenic disease can yield novel insights into both disease physiology and the ageing process itself.

## Introduction

The skeletal muscle channelopathies are a group of disorders whose manifestations range from flaccid paralysis to myotonia: They are broadly divided into the periodic paralyses (PP) and the non‐dystrophic myotonias.^1^ The PP include hyperkalaemic PP (HyperPP), hypokalaemic PP (HypoPP) and Andersen–Tawil syndrome. All these conditions are caused by dominant mutations in skeletal muscle ion channels that predispose to prolonged depolarization of the muscle membrane. In some circumstances, this increases the propensity to activate voltage‐gated sodium channels and trigger action potentials, resulting in a hyperexcitable membrane that clinically manifests as myotonia. However, regardless of the presence of preceding hyperexcitability, excessive depolarization can lead to inactivation of both normal and mutant sodium channels, rendering the muscle inexcitable.^2^, ^3^ This manifests as episodes of PP that usually occur in association with high or low serum potassium.^1^

In patients with PP, early in the disease course, muscle strength is reported to be normal in‐between attacks of paralysis. However, an unexplained but consistent clinical feature is that around the age of 40, a second clinical phase is observed, in which attack severity declines and severe, fixed and often disabling weakness develops.^4^, ^5^, ^6^, ^7^, ^8^, ^9^ It is unclear why dysfunction of implicated ion channels exhibits this biphasic natural history.

It is notable that in addition to being the age of phenotypic shift in PP patients, 40 is also the age at which even in healthy adults, optimal motor performance begins to decline,^10^ muscle mass decreases,^11^ grip strength reduces^12^ and mitochondrial abnormalities begin to be accepted as within the normal range for age on muscle biopsy.^13^ This suggests the possibility that age‐related change may contribute to the phenotype transition in PP.

**Figure 1:**
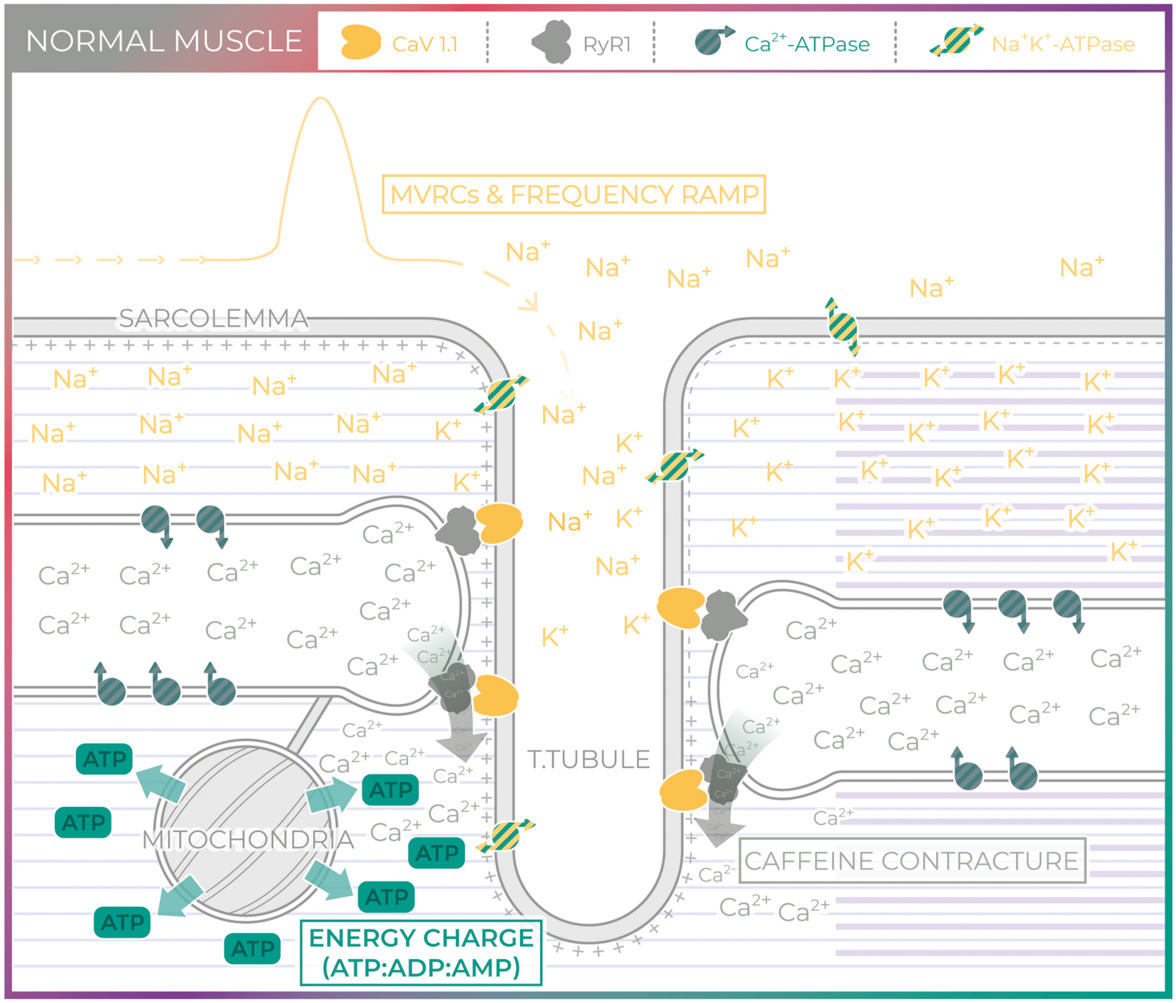
Normal muscle. Skeletal muscle excitability involves the initiation of an action potential at the neuromuscular junction (not shown) and subsequent propagation of an action potential along the sarcolemma and into the T‐tubules. Muscle velocity recovery cycles (MVRCs) and frequency ramp provide an indirect measurement of muscle excitability by examining changes in conduction velocity. Excitation–contraction coupling links muscle excitability to muscle contraction and requires depolarization of the T‐tubule voltage‐gated calcium channel (CaV1.1, yellow). Activation of CaV1.1 physically activates the sarcoplasmic reticulum (SR) calcium release channel (RyR1, grey), a process known as excitation–contraction coupling. Caffeine application bypasses excitability to act on RyR1 directly. Calcium released through RyR1 not only triggers calcium‐induced muscle contraction but also stimulates mitochondrial ATP synthesis. The energy charge is the ratio of ATP to ADP and AMP and reflects the metabolic status of the tissue. ATP is critical for ionic homeostasis, for example, to maintain skeletal muscle resting membrane potential via the Na⁺/K⁺‐ATPase (green and yellow stripes) and SR calcium stores via the SR Ca^2+^ ATPase (grey and green stripes). ATP is also critical for myosin–actin cross‐bridge cycling.

The Draggen mouse model of HyperPP carries an *SCN4A* gain‐of‐function mutation (I582V) in the skeletal muscle voltage‐gated sodium channel (Nav1.4), equivalent to that found in a patient with PP and myotonia (I588V).^14^ This mutation is located within the S1 segment of the second domain of NaV1.4. Heterozygote Draggen mice exhibit episodic attacks of hindlimb dragging, the number and severity of which can be very variable. However, an attack of weakness can be reliably induced *ex vivo* by exposure of Draggen muscle to a high‐potassium solution.^14^ The heterozygote Draggen mice also reproduce onset of fixed weakness with a progressive decline in grip strength from middle age as well as classic histological features of PP myopathy on muscle biopsy.^14^ Therefore, in this study, Draggen mice were used to characterize changes in ageing muscle and compared with ‘normal’ ageing observed in muscle from wild‐type (WT) littermates (*Figure* *1* **)**.

## Methods

### Animals

Male heterozygous Draggen mice (three generations of backcross onto C57Bl/6J)^14^ were bred with C57/BL 6J female mice. Only male mice were used in these studies as there is incomplete penetrance in females.^14^ Mice were fed *ad libitum* and housed according to home office guidelines. All experiments were performed in accordance with the ethical standards laid down in the 1964 Declaration of Helsinki and its later amendments. Experiments were carried out under licence from the UK Home Office (Scientific Procedures Act 1986) and following approval by the UCL Institute of Neurology Animal Welfare Ethical Review Panel. Some animals had a plastic flying saucer running wheel added to their cage from middle age to assess the effects of voluntary exercise on age‐related loss of muscle mass. Data from this group of animals were only included in the analysis of histology (*Figure* *6*) and weight (*Figure* *7*). In both cases, the values obtained from animals with access to a running wheel are given and are compared with values from data from animals without access to a running wheel.

### Experimental protocol

*In vivo* muscle velocity recovery cycles (MVRCs) and frequency ramp recordings were performed under terminal isoflurane anaesthesia on triceps brachii and tibialis anterior (TA) muscles bilaterally and were followed by dissection of TA and soleus or gastrosoleus. TA was then used for assessment of weight and adenylate energy charge. Soleus was used for functional testing (muscle tension testing followed by caffeine contracture or the potassium‐induced weakness assay), for Western blot or (when combined with gastrocnemius) for histological analysis.

MVRCs were not technically possible in soleus as it is a deep, thin muscle, and therefore, in a live and intact animal, we could not be certain we were recording from soleus. We chose TA for MVRCs as it is easily accessible and has been shown to develop the characteristic histological features of PP myopathy in aged Draggen mice.^14^ The mouse TA is composed predominantly (90%) of fast myofibres (Types 2X and 2B) compared with soleus that is ~90% slow myofibres (Types 1 and 2A).^15^ It was not possible to perform the potassium‐induced weakness assay in TA as it cannot be isolated with both tendons intact and therefore is not suitable for *ex vivo* muscle tension measurement.

We chose soleus for the *ex vivo* potassium‐induced weakness assay for several reasons. Firstly, this is because soleus is the mouse muscle that is most similar to human skeletal muscle in terms of gene expression and fibre‐typing.^16^ Secondly, this is because it is more susceptible to potassium‐induced weakness than the other muscle well suited to *ex vivo* muscle tension measurements, fast‐twitch glycolytic extensor digitorum longus.^17^ Thirdly, as HyperPP mouse muscle is known to switch towards a more oxidative fibre type from around 12 weeks of age^14^, ^18^ and this may alter sensitivity to potassium‐induced weakness,^16^ we chose a muscle that is already oxidative to reduce any potential contribution of fibre‐type transformation.

### MVRCs and frequency ramp recordings *in vivo*

Isoflurane anaesthesia was induced in an anaesthetic chamber. After induction, the mouse was placed on its back on a heat mat and anaesthesia maintained via a nose cone (*Figure* *S1*). As MVRCs have not been reported in rodents, they were performed as described previously for humans.^19^, ^20^, ^21^, ^22^ Recordings were performed *in vivo* on either TA (*Figure* *S1* *A*) or triceps (*Figure* *S1* *B*). The signal was amplified (gain 1000, bandwidth 50 Hz to 2 kHz) and digitized (NI DAQ) using a sampling rate of 20 kHz. The electrodes were adjusted to obtain a stable negative peak response with a stimulus of 3–10 mA. Stimulation and recording were controlled by QTRAC software using the M3REC3.QRP protocol. Surface temperature over the muscle was measured at the end of the recording either using an infrared thermometer or by applying a glass thermometer to the skin overlying the muscle. MVRCs were recorded with 1, 2 and 5 conditioning stimuli all separated by 10‐ms intervals. Test stimuli were delivered every 2 s. The inter‐stimulus interval between the last conditioning stimulus and the test stimulus varied from 1000 to 1.4 ms in 34 steps in an approximately geometric series (specifically 1000, 900, 800, 700, 600, 500, 450, 400, 350, 300, 260, 220, 180, 140, 110, 89, 71, 56, 45, 35, 28, 22, 18, 14, 11, 8.9, 7.1, 5.6, 4.5, 3.5, 2.8, 2.2, 1.8 and 1.4 ms).^19^

A 30‐Hz frequency ramp was performed as described for humans^19^ as it has not been reported for mice previously. The test stimulus was preceded by a 1 second train of stimuli at a frequency that was increased by 1 Hz on successive 2‐s cycles from 1 to 30 Hz. The average stimulation rate was therefore increased from 0.5 to 15.5 Hz over 1 min. Stimulus cycles with the test stimulus alone were recorded before (10 cycles at 0.5 Hz), during the ramp and for a further 30 s after the end of the ramp.^19^

### Muscle force measurements on soleus muscle *ex vivo*

The soleus muscle was dissected while the mouse remained under isoflurane anaesthesia and placed in the centre well of a three‐well custom‐made chamber designed by Scientific Systems Design Inc. and purchased via Digitimer. The two side wells were used to pre‐oxygenate and warm exchange solutions prior to application. Fluid in all three chambers was maintained at 30°C (as this was the temperature recorded from the surface of exposed soleus *in situ* using an infrared thermometer) and continuously bubbled with 95% O_2_, 5% CO_2_. The proximal muscle tendon was tied to a steel bar within the muscle chamber, and the distal tendon was tied to an isometric force transducer (Dynamometer UFI Devices). Tetanic stimuli (200‐ms train duration) were delivered using square wave pulses at supramaximal intensity applied via platinum wires positioned either side of the muscle. Muscles were adjusted to their optimal preload length to produce maximal tetanic contraction. Isometric contractile responses were recorded using a pen recorder (Lectromed MultiTrace 2) and digitized with PicoScope PC Oscilloscope 4424.

The potassium‐induced weakness assay was performed as described previously for the M1592V HyperPP mouse model, with concomitant changes in calcium included as this exacerbates the difference between mutant and WT mice.^17^, ^18^, ^23^, ^24^ The baseline bath solution contained NaCl 118 mmol; KCl 4.75 mmol; MgSO_4_ 1.18 mmol; CaCl_2_ 2.54 mmol; NaH_2_PO_4_ 1.18 mmol; glucose 10 mmol; NaHCO_3_ 24.8 mmol. The high‐potassium, low‐calcium solution differed from the baseline bath solution as follows: NaCl 113 mmol; KCl 10 mmol; MgCl_2_ 1.04 mmol; CaCl_2_ 1.3 mmol. The high‐calcium recovery solution contained the same as the baseline solution except CaCl_2_ was increased to 4 mmol. Muscles were not exposed to insulin or curare. The potassium‐induced weakness assay was only performed in muscles that had 10 min of stable (within 1 g) baseline force measurements. Tetanic force was measured by 50 V stimulation at 100 Hz (train duration 200 ms) every 2 min during baseline and high‐potassium solutions and at 2, 5, 10, 15 and 20 min during high‐calcium recovery solution.

To look for evidence of excitation–contraction uncoupling, caffeine contracture and tetanic force were examined as described previously.^25^ The force elicited from exposure to 50 mM caffeine was compared with the baseline tetanic force measured prior to caffeine application.

PicoScope 6 software was used to store and analyse the data. Muscles that had a baseline maximal tetanic force that was significantly weaker (>35% difference) than the contralateral muscle exposed to the same conditions were excluded as likely artefactual recordings secondary to injury. Muscles that had a caffeine contracture force/tetanic force ratio of >40% were excluded as this is associated with eccentric injury.^25^

### Histology

Soleus muscles from young, middle‐aged and old (age range 13–103 weeks) adult male WT and Draggen mice were dissected in combination with gastrocnemius under isoflurane anaesthesia, snap‐frozen in isopentane, and 10‐μm sections were cut in a cryostat. Sections were stained using haemotoxylin and eosin (H&E), cytochrome oxidase (COX) and succinate dehydrogenase (SDH) using standard protocols.^26^ Semi‐quantitative analysis of soleus was performed, assessing structural pathology, COX‐negative fibres and fibre typing, while blinded to animal age and genotype. Cores were defined light microscopically as lesions within myofibres characterized by loss of oxidative staining.^27^

### Immunoblot analysis to quantify protein levels

Soleus muscles were dissected and immediately flash‐frozen. To extract proteins, muscles were placed in lysis buffer [7 mL of sample buffer (Tris HCl 75 mM, pH 6.8, SDS 1%) and one tablet of Roche Complete Mini Protease inhibitor (14583920) in lysing matrix M tubes (MPBio)]. Samples were homogenized using the MP FastPrep‐24 homogenizer, spun at 4°C for 10 min at 10,000 rpm, and concentrations determined using Bio‐Rad DC protein quantification assay. Samples (20 μg sample protein diluted to make a total volume of 20 μL) were run for 2 h at 4°C on NuPAGE Tris‐Acetate 3–8% gels followed by wet transfer at 30 V for 5–6 h at 4°C onto a nitrocellulose membrane. Following transfer, membranes were stained with Ponceau to measure total protein. Membrane was incubated in TBS with 10% milk overnight at 4°C, then incubated with anti‐RyR1 (abcam 2868) diluted 1:1000 in TBST (TBS with 0.05% Tween20) for 1 h at room temperature and then washed three times for 10 min in TBST before incubation with an HRP conjugated secondary antibody at a dilution of 1:4000 (Santa Cruz sc‐2005#A2216 goat anti‐mouse) for 1 h at room temperature. After secondary antibody incubation, membranes were washed three times in TBS for 10 min and images taken using ChemiDoc MP imager.

### Energy charge measured by high‐pressure liquid chromatography

To extract the adenine nucleotides, TA muscle samples that had been immediately flash‐frozen in liquid nitrogen following dissection were weighed and homogenized in 0.5 mL ice‐cold 1.0 M perchloric acid, 250 μL of the homogenate was neutralized with 200 μL 0.5 M KHCO_3_ in 1 M KOH. The precipitated proteins and potassium perchlorate produced were removed by centrifugation at 13.000× *g* for 5 min, and the clear supernatants stored at −20°C until derivatization. 100 μL of 1.0 M sodium acetate (pH 4.5) and 20 μL 4 M chloroacetaldehyde were mixed with 100 μL of the neutralized extract and heated at 60°C for 40 min. After the incubation, samples were placed on ice for 5 min to cool and halt the reaction. Subsequently, 20 μL of the cooled derivatized sample was analysed by high‐pressure liquid chromatography (HPLC) using a C18 reversed‐phase column (Hypersil 5 ODS 4.6 × 150 mm, 3 μm) at a flow rate of 0.8 mL/min using a gradient from 100% 0.2 M KH_2_PO_4_, pH 5.0 to 98.9% 0.2 M KH_2_PO_4_, pH 5.0 1.1% acetonitrile over 31 min. Ethenoadenine nucleotides were determined by fluorescence detection at excitation/emission spectra pairs of 290_ex_/415_em_ nm.^28^ Peaks corresponding to the retention times for ATP, ADP and AMP were integrated, and peak areas obtained were used to calculate AEC using the equation

$$\mathrm{AEC}=\left(\left[\mathrm{ATP}\right]+0.5\left[\mathrm{ADP}\right]\right)∕\left(\left[\mathrm{ATP}\right]+\left[\mathrm{ADP}\right]+\left[\mathrm{AMP}\right]\right)$$

### Statistics

Firstly, a normality test was performed. Where there were two groups for comparison that passed the normality test, a student's *t*‐test was performed. This was usually with Welch correction as there were often differences in group size. When there were two groups and for one or more normality was rejected, either Welch rank test (MVRC or frequency ramp data) or Mann–Whitney U test (other data) was performed.

For three or more groups, when the primary question was whether a significant difference between groups and not interaction between factors was present, either a one‐way analysis of variance (ANOVA) or a Kruskal–Wallis ANOVA was performed depending on normality. Post hoc Tukey testing was performed when the ANOVA result was significantly different (*P* = <0.05 unless otherwise specified). If the primary question was whether there was interaction between factors, a two‐way ANOVA was performed.

Finally, when categorical data were classified in two different ways (e.g. specific histological feature observed on histology of either Draggen or WT muscle), a two tailed Fisher's exact test was used (http://vassarstats.net/) to examine the significance of any association between the two kinds of classification.

All data are represented as mean ± standard error of the mean (SEM) unless otherwise stated. As MVRCs and frequency ramp analysis involve examining multiple parameters simultaneously, an increased threshold for statistical significance of *P* < 0.01 was applied.

## Results

### Resistance to potassium‐induced weakness and onset of fixed weakness occurs in Draggen mice with age

**Figure 2:**
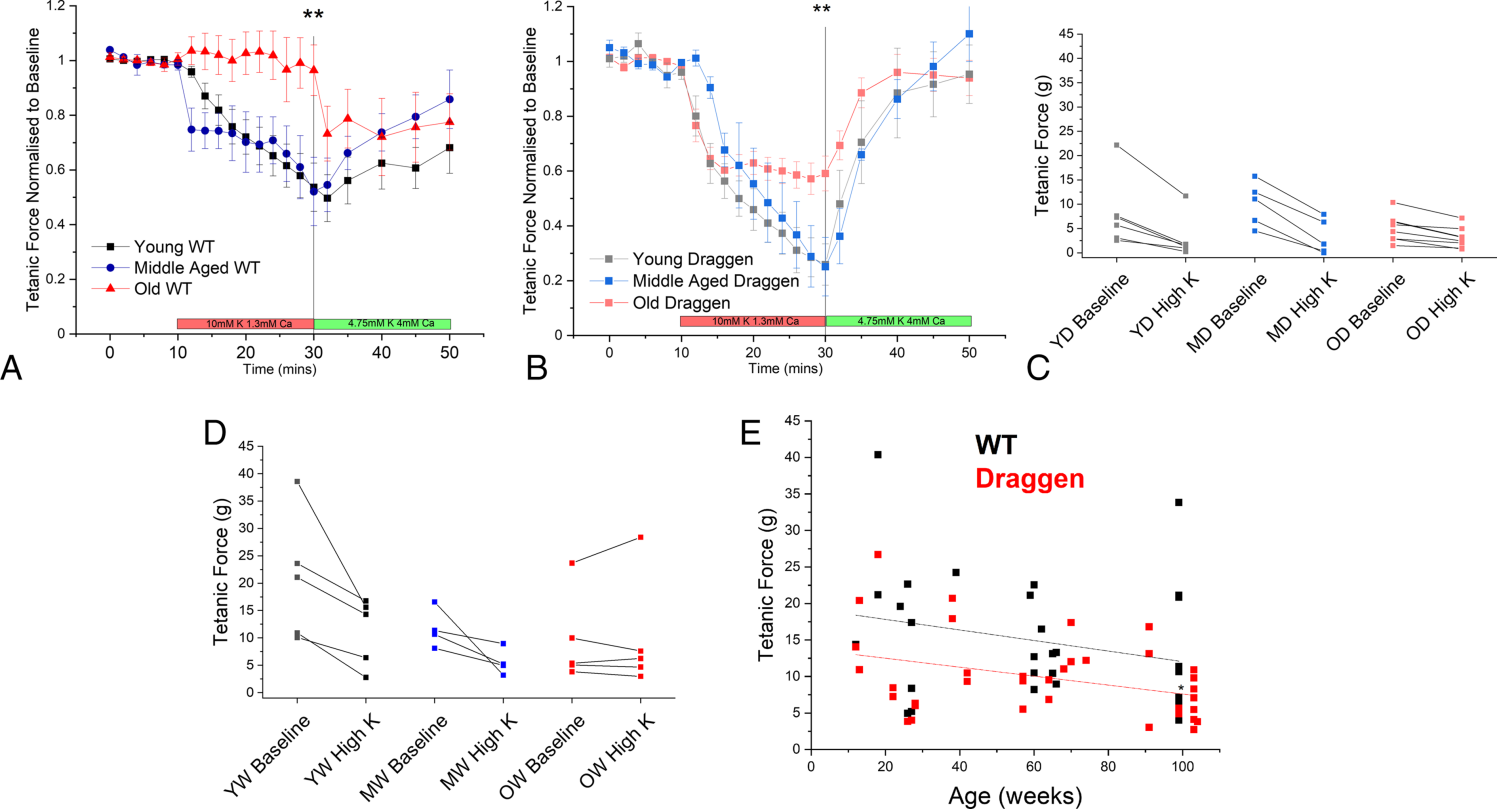
Changes in potassium‐induced weakness and muscle force with age. (A) Potassium‐induced weakness assay for young (black, *n* = 5) vs. middle‐aged (blue, *n* = 4) vs. old (red, *n* = 5) WT soleus muscles. (B) Potassium‐induced weakness assay for young (grey, *n* = 5) vs. middle‐aged (light blue, *n* = 5) vs. old (light red, *n* = 8) Draggen soleus muscles. (C) Absolute tetanic force at baseline and after 20‐min exposure to high‐potassium solution. Results are from individual young (YW), middle‐aged (MW) and old (OW) WT soleus. (D) Absolute tetanic force at baseline and after 20‐min exposure to high‐potassium solution. Results are from individual young (YD), middle‐aged (MD) and old (OD) Draggen soleus. (E) WT and Draggen soleus baseline tetanic force according to age (up to 104 weeks). *P < 0.05, **P < 0.01.

Isolated soleus muscles from young (13–26 weeks), middle‐aged (55–75 weeks) and old (95–104 weeks) WT (C57Bl/6J) mice and their Draggen littermates were used. A significantly larger reduction in muscle force occurred during the potassium‐induced weakness assay in Draggen compared with WT muscle in all age groups (*P* = 0.00005; *Figure* *2* *A* and *2* *B*). This was as described previously for 60‐week‐old Draggen EDL.^14^

There were no significant differences between young and middle‐aged groups for WT or Draggen mice. However, muscles from *both* old WT (*P* = 0.009) *and* old Draggen (*P* = 0.007) mice were significantly more resistant to potassium‐induced weakness than their respective groups of young and middle‐aged mice (*Figure* *2* *A*, *P* = 0.01 old vs. young WT, *P* = 0.02 old vs. middle‐aged WT; *Figure* *2* *B*, *P* = 0.02 old vs. young Draggen, 0.01 old vs. middle‐aged Draggen). Whereas old WT mice maintained force throughout exposure to the high‐potassium solution (*Figure* *2* *A*), old Draggen muscle still lost force but to a lesser degree than young or middle‐aged Draggen muscle (*Figure* *2* *B* **)**. The data were consistent for muscles with a range of baseline force (*Figure* *2* *C* and *2* *D*). In addition, it was noted that although force recovered fully in the Draggen mice following return to the recovery solution, the recovery was incomplete in WT mice (end of assay force − baseline force = −4.3 ± 1.7 g WT vs. − 0.1 ± 1.5 g Draggen, *P* = 0.01). In fact, old WT soleus actually lost force during exposure to the recovery solution, whereas young and middle‐aged WT muscle showed incomplete recovery (*Figure* *2* *A*) as has been observed previously.^18^

A decline in grip strength from middle age has been previously reported in Draggen mice, but soleus tetanic force was not measured.^14^ We found that there was a significant decline in Draggen soleus tetanic force with age (*P* = 0.03) although the rate of this decline did not differ from WT. However, the intercept of linear fits to force‐age data were significantly different between Draggen and WT soleus, suggesting reduced *ex vivo* force in young adult Draggen compared with WT soleus. Thus, Draggen mice showed reduced force compared with WT mice at baseline and in response to the potassium challenge, but *both* Draggen and WT mice showed increased resistance to potassium challenge with age.

### Changes in muscle excitability with age

MVRCs use changes in skeletal muscle conduction velocity to give an indirect measure of muscle excitability.^22^ They have been performed in humans and pigs but have not previously been reported in mice.^22^, ^29^ We performed MVRCs on TA and triceps muscles of WT and Draggen mice at the same three ages as in *Figure* *2*.

**Figure 3:**
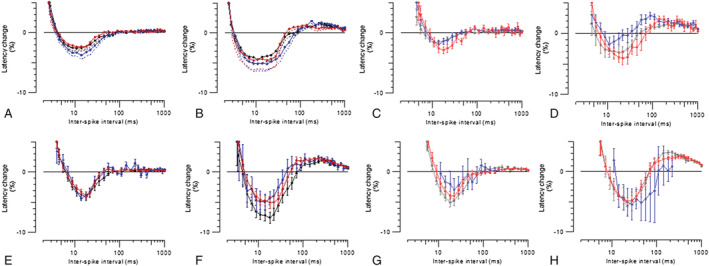
Muscle velocity recovery cycles (MVRCs) in TA and triceps muscles for WT and Draggen mice at three ages. (A) WT TA MVRCs in response to one conditioning stimulus. (B) WT TA MVRCs in response to five conditioning stimuli. (C) Draggen TA MVRCs in response to one conditioning stimulus. (D) Draggen TA MVRCs in response to five conditioning stimuli. (E) WT triceps MVRCs in response to one conditioning stimulus. (F) WT triceps MVRCs in response to five conditioning stimuli. (G) Draggen triceps MVRCs in response to one conditioning stimulus. (H) Draggen triceps MVRCs in response to five conditioning stimuli. WT TA young, *n* = 25; middle‐aged, *n* = 17, old, *n* = 17. Draggen TA young, *n* = 15; middle‐aged, *n* = 12 old, *n* = 12. WT triceps young, *n* = 23; middle‐aged, *n* = 6, old, *n* = 13. Draggen triceps young, *n* = 14; middle‐aged, *n* = 3, old, *n* = 10. Black = young, blue = middle‐aged, red = old. *n* = number of individual muscles per group.

There were significant differences in MVRCs between WT and Draggen mice at all ages (Supporting Information). MVRC data suggest that Draggen TA is depolarized relative to WT TA as the muscle relative refractory period is significantly longer (Supporting Information). This is consistent with microelectrode findings from the M1592V HyperPP muscle demonstrating depolarization relative to WT.^17^ Thus, MVRCs are sensitive enough to detect changes in skeletal muscle excitability and ion channel function in mice. However, despite this, there were no significant differences in MVRCs with age for either TA or triceps in WT or Draggen mice (*Figure* *3*). The fact that muscles of all ages respond similarly to single or multiple pre‐pulse stimulations suggests that both the potassium accumulation and the response to moderate activity‐induced potassium accumulation remain relatively constant throughout the life course.

**Figure 4:**
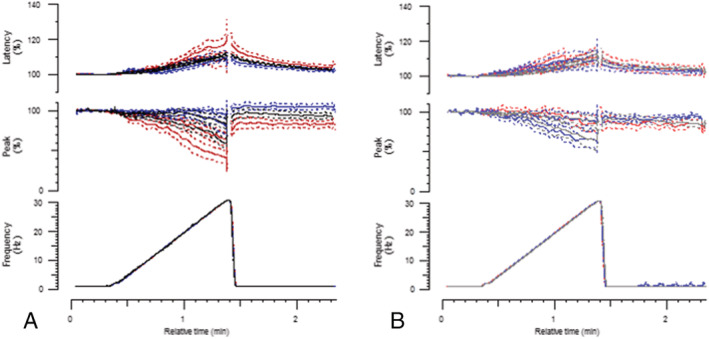
Response of WT and Draggen TA to *in vivo* 30‐Hz frequency ramp by age. (A) Response of WT TA to 30‐Hz frequency ramp. Young (black, *n* = 22), middle‐aged (blue, *n* = 13) and old (red, *n* = 14) mice. (B) Response of Draggen TA to 30‐Hz frequency ramp. Young (grey, *n* = 15), middle‐aged (light blue, *n* = 8) and old (light red, *n* = 9) mice. The change in latency (time from stimulus to peak of response) is plotted on the top row as a percentage change from baseline latency. The change in amplitude of response is plotted on the middle row as a percentage change from baseline amplitude of response. During the frequency ramp (shown on bottom row), the percentage change in response to both the first and last stimuli in the train are shown. The baseline is the mean latency and amplitude of response during the period of 0.5‐Hz stimulation that precedes the frequency ramp. ***P < 0.005, ****P < 0.0001.

Trains of action potentials increase the potassium concentration in the T‐tubules and around the muscle, and longer trains cause a greater increase in potassium.^30^ If the resistance to potassium‐induced weakness in old mice is because of resistance to potassium‐induced depolarization, then the amplitude of response should be better maintained during longer trains of action potentials in old compared with young or middle‐aged mice. However, rather than better maintenance of the amplitude of response, old WT TA had a significantly greater decrement in response to the first (*P* = 0.002) and last stimulus (*P* = 0.001) in a 30‐Hz train compared with younger WT muscle (*Figure* *4* *A*). The decrement in amplitude of response in old WT mice was accompanied by a progressive increase in latency (*Figure* *4* *A*) (slowing of conduction velocity), suggesting progressive depolarization and inactivation of NaV1.4 channels. The amplitude of response in old WT TA also failed to return to baseline and was significantly smaller as a percentage of its baseline 30 s after the ramp than middle‐aged WT TA (*P* = 0.000008) (*Figure* *4* *A*). There were no significant differences in the ramp recordings between young, middle‐aged and old Draggen TA (*Figure* *4* *B*). There were also no significant differences in ramp recordings with age for WT triceps or between young and old Draggen triceps (Supporting Information).

### Caffeine contracture force is significantly reduced in old Draggen soleus

Parameters controlling MVRCs do not appear to change with age (*Figure* *3*). Moreover, although old WT TA exhibits a reduction in the amplitude of response during high‐frequency stimulation (*Figure* *4*), old Draggen TA does not. This suggests that changes in parameters that lead to a reduction in frequency ramp response amplitude are not the cause for the fixed progressive weakness in Draggen muscle. As parameters controlling muscle excitability (in MVRCs) do not change significantly with age, the aetiology of the fixed progressive weakness in Draggen mice is likely to lie downstream of muscle excitability. To investigate excitation–contraction coupling, we studied excitation–contraction coupling using caffeine contracture force *ex vivo*. Caffeine bypasses muscle excitability and acts directly on ryanodine receptors (RyR1) to cause calcium release and muscle contraction (*Figure* *1*).

**Figure 5:**
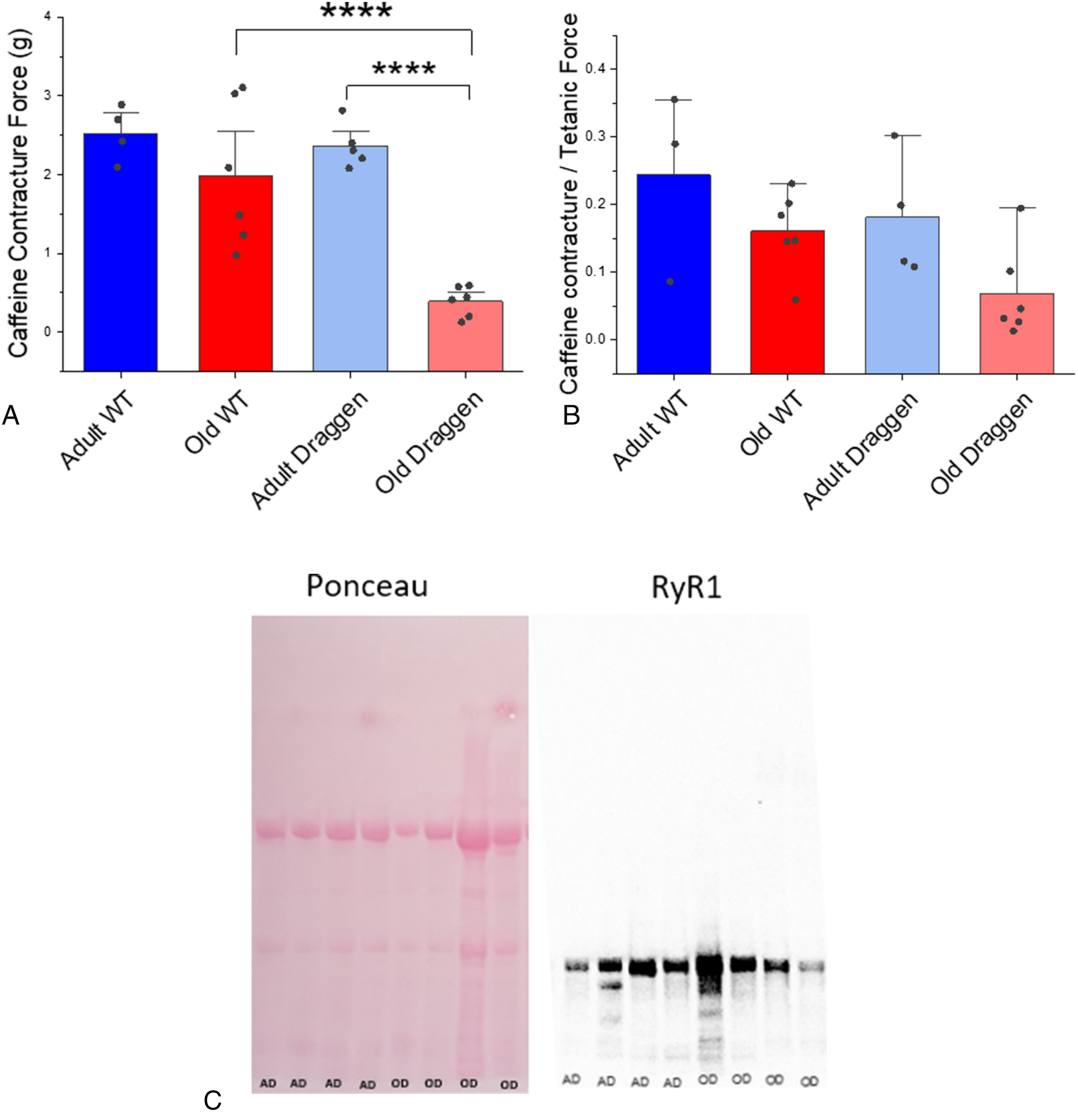
Caffeine contracture force of adult and old WT and Draggen soleus. (A) Caffeine contracture force compared for WT and Draggen mice at two ages: adult (13–42 weeks) and old (91–103 weeks). Individual data points are shown. (B) The ratio of caffeine contracture to tetanic force is compared for WT and Draggen mice at the same two ages as (A). Individual data points are shown. (C) Ponceau to compare total protein and Western blot to measure RyR1 expression in adult and old Draggen soleus (Abcam 2868 1:1000) AD = adult Draggen (18–68 weeks), OD = old Draggen (99–100 weeks); ****P < 0.001.

Caffeine contracture force was significantly reduced with age in Draggen but not WT soleus (*P* = 0.00005) (*Figure* *5* *A*). There was also a trend (*P* = 0.09) towards a *reduction* in the ratio between caffeine contracture force and tetanic force *ex vivo* in old Draggen soleus (*Figure* *5* *B*). An *increase* in this ratio would be expected in the event of failure of CaV1.1 to activate RyR1 (excitation–contraction uncoupling). Therefore, the reduced caffeine contracture of old Draggen muscle suggests impairment of RyR1 Ca^2+^ release, SR Ca^2+^ storage or Ca^2+^‐induced muscle contraction but not excitation–contraction uncoupling.

A reduction in RyR1 expression is one possible cause of reduced caffeine contracture force. However, band thickness on Western blots comparing adult (13–42 weeks, *n* = 4) and old (91–103 weeks, *n* = 4) Draggen soleus suggests that RyR1 protein levels were similar (*Figure* *5* *C*). This is in keeping with the reported lack of age‐related change in healthy muscle RyR1 expression in humans^31^, ^32^ and mice.^33^ This suggests that functional impairment in RyR1 calcium release rather than reduced levels of RyR1 protein accounts for the reduced caffeine contracture force in old Draggen soleus.

### Structural core pathology is prevalent in the muscle of Draggen mice

**Figure 6:**
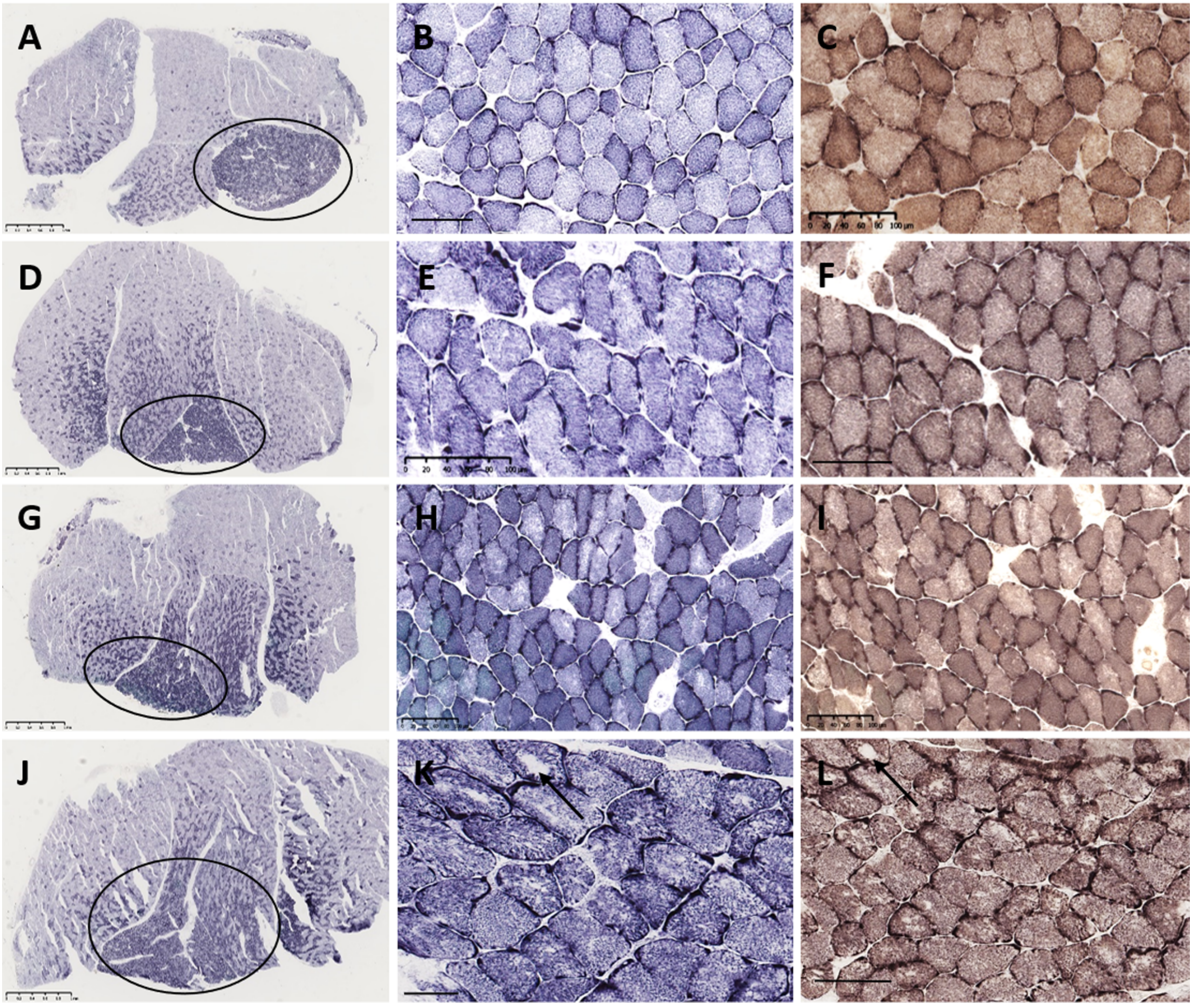
Soleus muscle pathology in young and ageing wild‐type and Draggen mice. Young wild‐type mouse (A–C). Young Draggen mouse (D–F). Ageing wild‐type mouse (G–I). Ageing Draggen mouse (J–L). Sections stained with NADH‐TR (A,B,D,E,G,H,J,K) and COX‐SDH (G,F,I,L). Scanning magnification view (A,D,G,J) in all animals shows a transverse section through the mid‐belly of the gastrocnemius–soleus muscle. The smaller oxidative, Type I fibre predominant soleus (circle) is present under the larger mixed‐fibre‐type gastrocnemius. In the young wild‐type (B,C) and young Draggen (E,F), the muscle architecture is normal. In the aged wild‐type mouse (H,I), there is subtle uneven oxidative staining in a proportion of fibres, but no overt pathology. In contrast, in the aged Draggen mouse (K,L), both show florid core pathology affecting several fibres, ranging from marked unevenness of oxidative staining, multicores, discrete small cores and occasionally well‐defined larger cores (K,L, arrow). Scale bar: A, D, G, J = 1 mm; *N* = 50 μm; B, C, E, F, H, I, K, L = 100 μm.

Cores and mini‐cores are seen in patients with myopathies due to mutation of RyR1. One of 10 WT soleus examined (age range 27–103 weeks, mean age 78 weeks) and 11 of the 16 Draggen soleus examined (age range 13–103 weeks, mean age 66 weeks) had cores and core‐like regions (*P* = 0.005, two‐tailed fisher exact test) (*Figure* *6*). This included animals with (*n* = 7 Draggen, *n* = 5 WT) and without (*n* = 8 WT, *n* = 9 Draggen) access to a voluntary running wheel.

The only WT animal with core pathology was a 62‐week‐old animal that did not have access to a voluntary running wheel. Six of 9 Draggen mice that did not have access to a voluntary running wheel had cores compared with five of seven Draggen mice with access to a voluntary running wheel. In those without access to a voluntary running wheel, cores were not seen in the youngest two animals (13 and 14 weeks) but were seen in a 27‐week‐old animal. In those with access to a voluntary running wheel, two of the three middle‐aged animals did not have cores, whereas all old animals did. When combined, the average age of Draggen soleus with cores was 80.6 ± 23.3 weeks (*n* = 11) vs. an average age of 36.4 ± 32 weeks without cores (*n* = 5). Cores were found in six out of the seven old (>97 weeks) Draggen soleus muscles vs. zero out of four old WT.

The finding of cores in many (six/seven) old Draggen but not WT soleus suggests that the reduced caffeine contracture force in old Draggen is associated with acquired RyR1 dysfunction. The observation of cores in a 27‐week‐old animal suggests that core formation may precede the reduction in caffeine contracture force in Draggen muscle.

Apart from the structural core pathology described above, there were no other overt myopathic or dystrophic changes, and no significant mitochondrial pathology in the ageing WT and Draggen mice.

### Draggen skeletal muscle energy homeostasis is impaired with age

**Figure 7:**
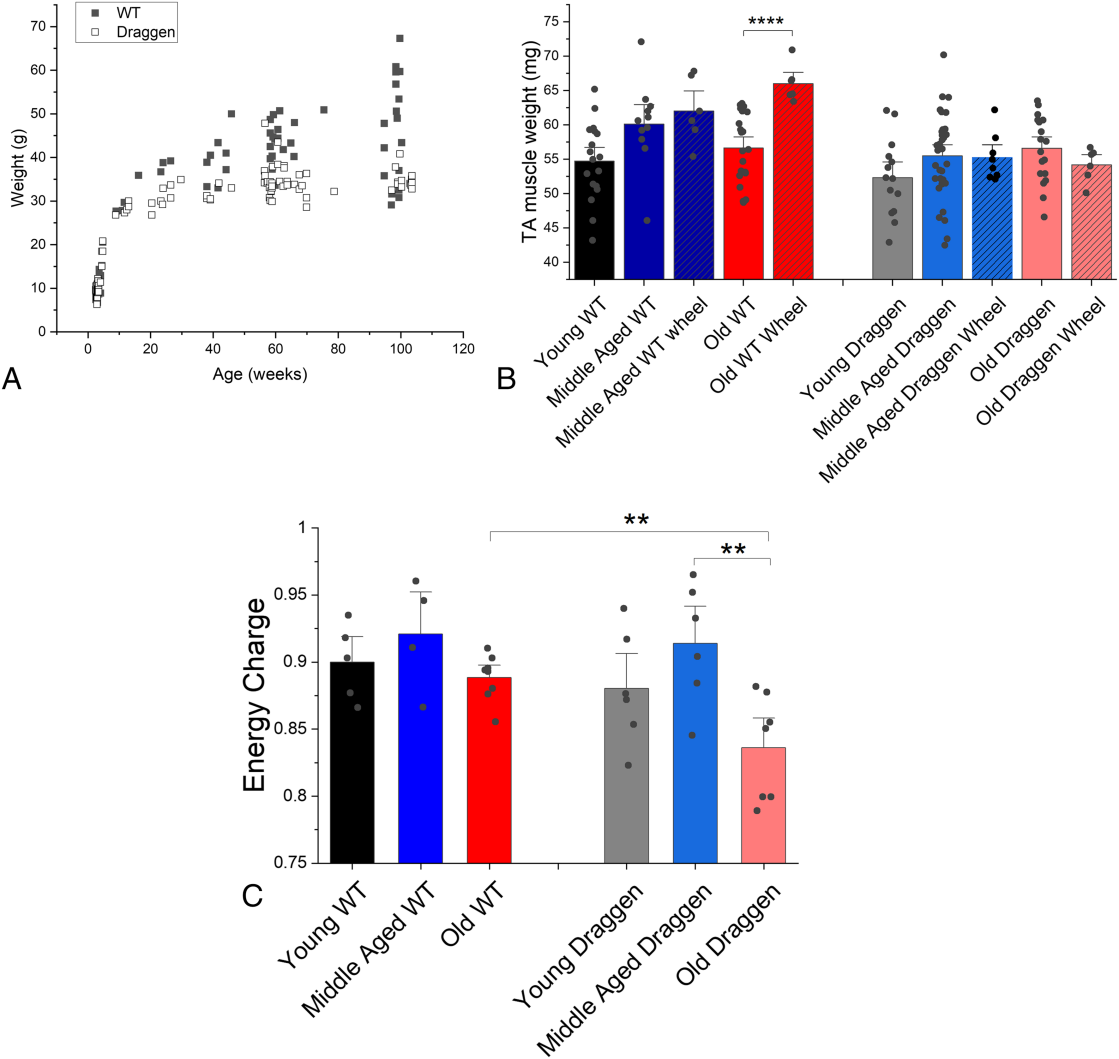
Evidence of impaired energy homeostasis in old Draggen muscle. (A) The body weights of WT (black square) and Draggen (empty circle) male mice measured at weaning (3 weeks) and at time of terminal experiment (13–104 weeks). (B) Weight of dissected TA muscle at time of terminal experiment in mice with and without access to a voluntary running wheel. Middle‐aged wheel = voluntary running wheel added to cage when mice aged 51–57 weeks and TA muscle dissected and weighed after 9–18 weeks (WT, *n* = 6 muscles) or after 24 weeks (Draggen, *n* = 6 muscles). Old wheel = voluntary running wheel added to the cage when mice aged 72–74 weeks and TA muscle was dissected and weighed after 25 weeks (*n* = 6 muscles for WT and Draggen). (C) The adenylate energy performed after MVRCs and 30‐Hz frequency ramp. **P < 0.01, ****P < 0.001.

Mouse models with acquired or genetic RyR1 dysfunction have impaired ATP production^34^ as a result of depolarized mitochondrial membranes.^34^, ^35^ Male Draggen mice are known to have increased energy expenditure compared with their male WT littermates. They exhibit reduced body weight due to fat loss, but muscle weight was conserved at ages up to 54 weeks.^14^ The reduced body weight without significant difference in muscle weight up to 54 weeks was confirmed in our study (*Figures* *7* *A* and *S2*).

However, if energy deficit increases with age, then the weight difference between Draggen and WT mice should also increase with age past 54 weeks. Consistent with this hypothesis, the mean weight difference was greatest in old mice (mean WT–mean Draggen weight was 0.8 g at weaning, 3.8 g between 9 and 30 weeks, 8.5 g between 38 and 79 weeks and 11.6 g between 95 and 104 weeks; *Figure* *7* *A*).

Access to a voluntary running wheel from middle age has been shown to prevent age‐related loss of muscle mass in WT mice.^36^ In keeping with these findings, the mean TA weight for old WT males with access to a running wheel from 72 to 74 weeks of age for 25 weeks was 66 mg (± 2.7 SD, *n* = 6 TA muscles) vs. 56 mg (± 5.1 SD, *n* = 11, TA muscles) for age‐matched WT males without access to a running wheel (*P* = 0.00002) (*Figure* *7* *B*). In contrast, in old Draggen mice, access to a voluntary running wheel from 71 weeks of age for 25 weeks led to a trend towards reduction in TA muscle weight (*Figure* *7* *B*). The mean TA weight of old Draggen mice with access to a running wheel was 54 mg ± 2.5 SD (*n* = 6 muscles) vs. 57 mg ± 4.8SD (*n* = 19 muscles**)** for age‐matched Draggen mice without access to a wheel (*Figure* *7* *B*; *P* = 0.09). This suggests that exercise in old age may have exacerbated the energy deficit of Draggen mice.

Activation of the catabolic or anabolic pathway is determined by the metabolic status of skeletal muscle. This is reflected by the adenylate energy charge, which measures the ratio of ATP to ADP and AMP. The energy charge performed after MVRCs and 30‐Hz frequency ramp stimulation was significantly lower in old Draggen TA than either old WT (*P* = 0.004) or middle‐aged Draggen TA (*P* = 0.006) (*Figure* *7* *C*). This indicates that the activity from MVRCs and 30‐Hz frequency ramp was enough to deplete ATP reserves in old Draggen but not adult Draggen or old WT muscle.

### Discussion

Our studies of changes in muscle function with age reveal that muscle force becomes more resistant to potassium‐induced weakness in *both* old WT and Draggen mice. The human age equivalents of the mouse age groups used are approximately 20–30, 43–50 and 65–70 years, respectively.^37^

Our data are consistent with the proposal that as muscle ages, it develops mechanisms to maintain force *despite* potassium‐induced sodium channel inactivation and sarcolemmal depolarization. This suggests that the reduction in attack severity observed in patients with PP may be part of the phenotype shift observed in normal human ageing.

In contrast, the age‐related muscle pathology was specific to Draggen muscle, indicating that it is caused, or at least accelerated by, chronic genetic ion channel dysfunction.

To our knowledge, this is the first time MVRCs have been reported in rodents. As evidenced by the significant differences in MVRC parameters from Draggen and WT TA (Supporting Information), like human MVRCs,^19^, ^20^, ^21^, ^22^ mouse MVRCs can detect changes in skeletal muscle excitability due to genetic ion channel dysfunction.

It was not technically possible to perform MVRCs in soleus. However, the lack of significant change in mouse MVRCs with age in either triceps or TA implies that increased resistance to potassium‐induced weakness is not due to universal changes in baseline muscle excitability. The fact that decrement in amplitude of response after a train of 30 stimuli was similar (Draggen TA, WT triceps) or even more pronounced (WT TA) in old compared with young or middle‐aged muscle suggests that the maintenance of tetanic force in old muscle subject to the potassium‐induced weakness assay is not due to a resistance to potassium‐induced sarcolemmal depolarization. Variation in extracellular K^+^, Cl^−^ and Na^+^ cannot underlie the increased resistance of old muscle to potassium‐induced weakness as extracellular ionic concentrations were constant. Therefore, the data are consistent with old muscle developing intrinsic mechanisms to maintain force, despite normal (or for old WT TA apparently increased) sodium channel inactivation and sarcolemmal depolarization.

The exact mechanism for the apparent resistance to potassium‐induced weakness with age remains to be determined. Our data suggest that force and excitability can be differentially regulated in old muscle and more specifically that reduced excitability may not translate into the expected reduction in force. Reviewing the literature on PP, previous studies have also provided evidence that force and excitability can be dissociated.^38^, ^39^, ^40^, ^41^, ^42^ In isolated human myofibres from a patient with HyperPP, reduction of pH restored force but not resting membrane potential during potassium‐induced weakness.^38^ In the HypoPP mouse models, acetazolamide was effective at preventing CMAP decrement*,* but did not prevent loss of muscle force during induced attacks.^39^, ^40^ In humans with HypoPP, action potential initiation and propagation failed *after* muscle twitch tension during an induced attack of paralysis,^42^ and in another study, the patient had regained sufficient muscle strength to flex their arm against gravity *before* direct electrical stimulation could elicit a muscle twitch.^41^ How force is maintained despite a depolarized resting membrane potential as observed in the Hyper PP muscle fibres, and why the maintenance of CMAP does not translate into maintenance of force in mice or humans with Hypo PP, remains unclear but may shed light on how healthy and PP old muscle can maintain tetanic force despite depolarization.

Baseline soleus tetanic force of Draggen mice showed significant decline with age (*P*= 0.03). The intercept (*P* = 0.006), but not the slope of a linear fit to force‐age data was different between Draggen and WT mice, suggesting lower baseline *ex vivo* tetanic force in Draggen mice (*Figure* *2* *E*). A lower baseline force for Draggen muscle was not detected in grip strength experiments *in vivo*, but grip strength did decline in Draggen mice after 60 weeks of age.^14^ As the baseline force decline was not detected *in vivo*, it is possible that the reduced baseline force in Draggen may be the result of increased sensitivity of Draggen muscle to the *ex vivo* conditions. A depolarization of resting membrane potential by approximately 20 mV has been reported when comparing the same mouse muscle resting membrane potential measured *in vivo* or *in situ* vs. *ex vivo*.^43^ As our MVRC data (see Supporting Information) suggest that Draggen muscle is relatively depolarized compared with WT muscle *in vivo*, an additional depolarization of this scale would potentially have a disproportionate effect on Draggen, compared with WT muscle force measured *ex vivo*.

**Figure 8:**
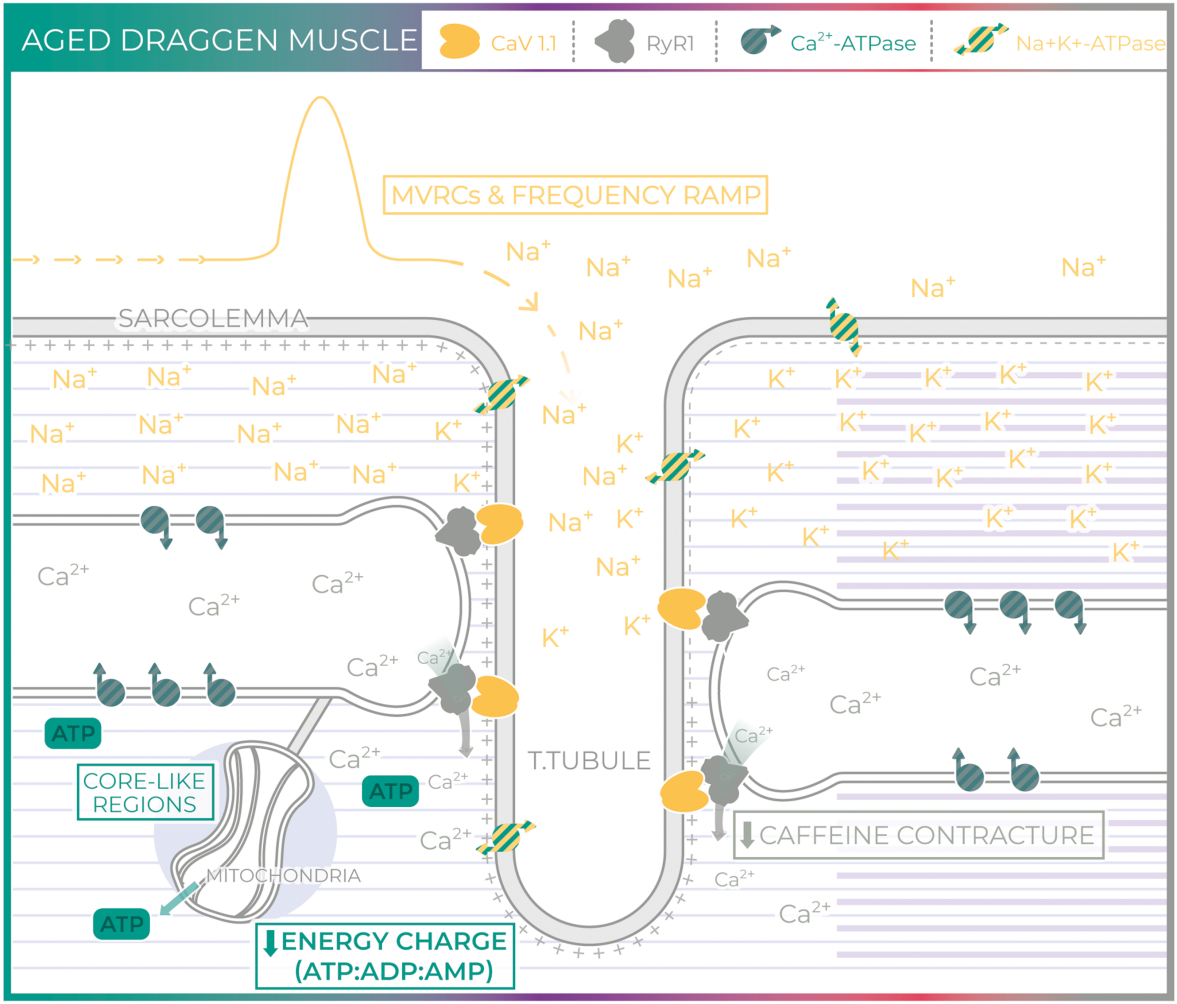
Aged Draggen muscle. In aged Draggen muscle, we found no evidence of a change in baseline muscle excitability with age (MVRCs and frequency ramp). However, caffeine contracture force and energy charge were significantly lower in old Draggen muscle, and core‐like regions were found in old Draggen but not WT muscle. Taken together, our data suggest that progressive acquired RyR1 dysfunction and associated impairment of energetic homeostasis are responsible for the fixed weakness in old Draggen mice.

Old age in Draggen muscle was associated with reduced caffeine contracture force (*Figure* *5* *A*), decreased energy charge (*Figure* *7* *D*) and structural core pathology (*Figures* *6* and *8*). Pathogenic mutations in at least eight genes have been associated with core myopathies in humans and occur most frequently due to mutations in *RYR1*, *TTN* and *MYH7*.^27^ Core‐like regions have also been reported in mouse models with impaired ATP production secondary to RyR1 mutation.^34^ Thus, acquired RyR1 dysfunction seems the most likely cause for the reduced caffeine contracture force, decreased energy charge and the structural core pathology observed in aged Draggen muscle (*Figure* *8*).

Acquired RyR1 dysfunction resulting in Ca^2+^ leak and consequent SR Ca^2+^ store depletion has been reported in normal aged mouse and human muscle.^31^, ^32^, ^35^, ^44^ In these studies, SR calcium concentration and release rather than caffeine contracture were measured. This difference in technique may explain why we failed to see any significant deficit in WT muscle caffeine contracture. However, given these observations in normal ageing, it seems likely that fixed progressive weakness in Draggen mice represents a form of accelerated ageing rather than a process specific to Draggen muscle.

Pertinent to these observations are conclusions from a detailed time course study of molecular changes across the life span in rats for various ageing tissue^45^ that showed the most prominent common pathway downregulated with ageing was related to mitochondrial oxidative phosphorylation and respiratory electron transport (in skeletal muscle, liver and kidney), consistent with the proposal that the mitochondria become less competent with age, depriving cells of critical supplies of ATP. Calcium release through RyR1 is known to stimulate mitochondrial ATP production.^46^ Thus, in normal ageing, the combination of impaired calcium release reducing the stimulus for mitochondrial ATP production and decreased mitochondrial oxidative capacity with age could form a double hit for ageing skeletal muscle. This would be expected to manifest earlier in male Draggen mice because of their increased energy requirements.^14^ As exercise would exacerbate this energy deficit by increasing energy requirement further, the observation that old Draggen mice were unable to mount an anabolic response to exercise is in keeping with this hypothesis (*Figure* *7* *D*).

This study reinforces the close links between membrane excitability, RyR1 and mitochondrial function (*Figures* *1* and *8*). The interconnected nature of RyR1 and mitochondrial function with membrane excitability is apparent in both humans and mice as evidenced by reports of humans with RyR1 mutations^47^ and mitochondrial DNA mutations^48^ that have a PP‐like phenotype; a patient with PP due to mutation in NaV1.4 that responds to treatment with coenzyme Q10^49^ and mice with an RyR1 mutation and core‐like regions on muscle biopsy that exhibit potassium responsive weakness similar to PP.^34^

One of the limitations of this study is that we did not measure the frequency of spontaneous attacks in Draggen mice and therefore cannot comment on whether reduction in the severity of potassium‐induced weakness is associated with a reduction in the frequency of spontaneous attacks. In addition, we limited the study to male mice because, as for humans with the condition, some female carriers are asymptomatic.^14^, ^50^ It is not known if a gender difference in the PP phenotype change with age exists, and so, future work should confirm that a similar phenomenon is observed in females and is associated with the same pathophysiology. Finally, as discussed in the methods, for technical reasons, we could not perform MVRCs on soleus nor the potassium‐induced weakness assay on TA. It is therefore possible that the resistance to potassium‐induced weakness with age is limited to soleus (composed mainly of oxidative fibres) and impairment of energy homeostasis to TA (composed mainly of glycolytic fibres),^15^ especially as there are complex effects of ageing on myofibre‐type differences in contractile and metabolic properties. This impact of myofibre type could be tested by using the fast twitch glycolytic extensor digitorum longus muscle (~90% Types 2X and 2A^15^) to measure changes in potassium‐induced weakness with age. However, as human muscles are predominantly oxidative and have been reported to be most similar to soleus transcriptomically,^16^ from a translational point of view, the value of this may be limited. Moreover, although energy charge and the effect of exercise on muscle weight were only measured in TA, the observations of reduced caffeine contracture force combined with core‐like structures in aged Draggen soleus provide independent evidence to suggest that oxidative phosphorylation is impaired.^34^, ^35^, ^46^

An increased energy expenditure and/or deficit in body weight has not, to our knowledge, been reported in humans with PP. However, PP is a rare disease, and it is likely that unless specifically looked for subtle difference in body weight may have been missed. A decrease in the ATP to ADP ratio has been observed for patients with myotonic dystrophy and associated with a small but significant reduction in mitochondrial function.^51^ This suggests that the increased energetic requirement of ion channel dysfunction may also affect human muscle but may not be associated with clear weight discrepancies. Like the study in myotonic dystrophy, magnetic resonance spectroscopy could be used to confirm an ATP deficit in older humans with PP. If present, muscle biopsies to specifically look for core pathology should be considered. Structural pathology consistent with cores in our opinion (originally reported as target lesions) has been reported in one kindred with normokalaemic PP, which is believed to be within the spectrum of HyperPP.^52^, ^53^ However, they have not been a common finding in PP. In this case, the gastrocnemius was biopsied, and the biopsy was taken after a very prolonged attack after which the patient had not fully recovered force.^53^ Gastrocnemius is one of the most commonly and severely affected muscles on MRI of HyperPP patients.^54^ However, our clinical practice, as for many other centres in Europe and the United States, is to perform biopsies of quadriceps, deltoids or biceps. This, along with the fact that patients tend to be biopsied early in the course of the disease, may explain why cores have not to date been a prominent feature reported for PP patients.

In summary, our data demonstrate that phenotype transition with age also occurs in the Draggen mouse model of HyperPP. Though intrinsic muscle ageing protects against potassium‐induced weakness in Draggen mice, it is also associated with impaired SR Ca^2+^ release, a process that seems to be accelerated in Draggen muscle. Thus, ageing can modify the clinical effect of genetic mutation. In conclusion, this work provides a replicable example of how studying phenotype change with age in monogenic disease can yield novel insight into both disease physiology and the ageing process itself.

## Author contributions

KS: Designing research studies, conducting experiments, acquiring data, analysing data, providing reagents, writing and revising the manuscript. SVT: Designing research studies, analysing data and revising manuscript. RM: Designing research studies, providing reagents, analysing data and revising manuscript. RP: Analysing data and revising manuscript. MO: Conducting experiments and analysing data. SE: Providing reagents, analysing data and revising manuscript. AAS: Revising manuscript. MDG: Revising manuscript. EM: Designing research studies, analysing data and revising manuscript. LG: Designing research studies, providing reagents, analysing data and revising manuscript. MGH: Designing research studies, analysing data and revising manuscript.

## Conflict of interest

The authors have declared that no conflict of interest exists.

## Supporting information


**Figure S1.** Experimental setup for MVRCs in mouse muscle. a. Experimental set up for TA b. Experimental set up for triceps. In both A and B a monopolar stimulating needle electrode (28G TECA, Viasys Healthcare Madison, Wisconsin) was inserted into the distal muscle. A reference anode was inserted slightly above and lateral to the monopolar stimulating electrode. The reference anode consisted of a 27G hollow bore disposable steel needle attached to reference anode lead with crocodile clip. Stimuli consisting of 0.05 ms rectangular current pulses were delivered. Muscle activity was recorded with a concentric needle electrode (disposable 30G concentric EMG needle, TECA) inserted into the proximal end of the muscle. A ground electrode was inserted under the skin in the axilla. The ground electrode consisted of a 27G hollow bore disposable steel needle that was bent to make it easier to insert under the skin and attached to crocodile clip on the ground cable.

**Figure S2.** Weight of dissected soleus muscle at time of terminal experiment in mice without access to a voluntary running wheel.

**Data S1.** Supporting Information

## References

[1] Suetterlin K , Männikkö R , Hanna MG . Muscle channelopathies: recent advances in genetics, pathophysiology and therapy. Curr Opin Neurol 2014;27:583–590. 25188014 10.1097/WCO.0000000000000127

[2] Cannon SC , Brown RH Jr , Corey DP . A sodium channel defect in hyperkalemic periodic paralysis: potassium‐induced failure of inactivation. Neuron 1991;6:619–626. 1849724 10.1016/0896-6273(91)90064-7

[3] Sokolov S , Scheuer T , Catterall WA . Gating pore current in an inherited ion channelopathy. Nature 2007;446:76–78. 17330043 10.1038/nature05598

[4] Miller TM , Dias da Silva MR , Miller HA , Kwiecinski H , Mendell JR , Tawil R , et al. Correlating phenotype and genotype in the periodic paralyses. Neurology 2004;63:1647–1655. 15534250 10.1212/01.wnl.0000143383.91137.00

[5] Links TP , Zwarts MJ , Wilmink JT , Molenaar WM , Oosterhuis HJ . Permanent muscle weakness in familial hypokalaemic periodic paralysis. Clinical, radiological and pathological aspects. Brain J Neurol 1990;113:1873–1889. 10.1093/brain/113.6.1873 2276049

[6] Sternberg D , Maisonobe T , Jurkat‐Rott K , Nicole S , Launay E , Chauveau D , et al. Hypokalaemic periodic paralysis type 2 caused by mutations at codon 672 in the muscle sodium channel gene SCN4A. Brain J Neurol 2001;124:1091–1099. 10.1093/brain/124.6.1091 11353725

[7] Gamstorp I . Adynamia episodica hereditaria. Acta Genet Stat Med 1957;7:325–328. 13469175 10.1159/000150999

[8] Charles G , Zheng C , Lehmann‐Horn F , Jurkat‐Rott K , Levitt J . Characterization of hyperkalemic periodic paralysis: a survey of genetically diagnosed individuals. J Neurol 2013;260:2606–2613. 23884711 10.1007/s00415-013-7025-9 PMC3824221

[9] Ptacek LJ , Trimmer JS , Agnew WS , Roberts JW , Petajan JH , Leppert M . Paramyotonia congenita and hyperkalemic periodic paralysis map to the same sodium‐channel gene locus. Am J Hum Genet 1991;49:851–854. 1654742 PMC1683172

[10] Schott JM . The neurology of ageing: what is normal? Pract Neurol 2017;17:172–182. 28455389 10.1136/practneurol-2016-001566

[11] Smith GI , Mittendorfer B . Sexual dimorphism in skeletal muscle protein turnover. J Appl Physiol 2016;120:674–682. 26702024 10.1152/japplphysiol.00625.2015 PMC4796180

[12] Dodds RM , Syddall HE , Cooper R , Benzeval M , Deary IJ , Dennison EM , et al. Grip strength across the life course: normative data from twelve British studies. PLoS One 2014;9:e113637. 25474696 10.1371/journal.pone.0113637 PMC4256164

[13] Pesce V , Cormio A , Fracasso F , Vecchiet J , Felzani G , Lezza AMS , et al. Age‐related mitochondrial genotypic and phenotypic alterations in human skeletal muscle. Free Radic Biol Med 2001;30:1223–1233. 11368920 10.1016/s0891-5849(01)00517-2

[14] Corrochano S , Männikkö R , Joyce PI , McGoldrick P , Wettstein J , Lassi G , et al. Novel mutations in human and mouse SCN4A implicate AMPK in myotonia and periodic paralysis. Brain 2014;137:3171–3185. 25348630 10.1093/brain/awu292 PMC4240299

[15] Kammoun M , Cassar‐Malek I , Meunier B , Picard B . A simplified immunohistochemical classification of skeletal muscle fibres in mouse. Eur J Histochem EJH 2014;58:2254. 24998919 10.4081/ejh.2014.2254 PMC4083319

[16] Kho AT , Kang PB , Kohane IS , Kunkel LM . Transcriptome‐scale similarities between mouse and human skeletal muscles with normal and myopathic phenotypes. BMC Musculoskelet Disord 2006;7:1–9. 16522209 10.1186/1471-2474-7-23 PMC1525166

[17] Ammar T , Lin W , Higgins A , Hayward LJ , Renaud J‐M . Understanding the physiology of the asymptomatic diaphragm of the M1592V hyperkalemic periodic paralysis mouse. J Gen Physiol 2015;146:509–525. 26621775 10.1085/jgp.201511476 PMC4664826

[18] Hayward LJ , Kim JS , Lee M‐Y , Zhou H , Kim JW , Misra K , et al. Targeted mutation of mouse skeletal muscle sodium channel produces myotonia and potassium‐sensitive weakness. J Clin Invest 2008;118:1437–1449. 18317596 10.1172/JCI32638 PMC2260907

[19] Tan SV , Z'Graggen WJ , Boërio D , Rayan DR , Norwood F , Ruddy D , et al. Chloride channels in myotonia congenita assessed by velocity recovery cycles. Muscle Nerve 2014;49:845–857. 24037712 10.1002/mus.24069

[20] Tan SV , Z'Graggen WJ , Hanna MG , Bostock H . *In vivo* assessment of muscle membrane properties in the sodium channel myotonias. Muscle Nerve 2017;57:586–594. 28877545 10.1002/mus.25956 PMC5839928

[21] Tan SV , Z'graggen WJ , Boërio D , Rayan DLR , Howard R , Hanna MG , et al. Membrane dysfunction in Andersen‐Tawil syndrome assessed by velocity recovery cycles. Muscle Nerve 2012;46:193–203. 22806368 10.1002/mus.23293

[22] Z'Graggen WJ , Bostock H . Velocity recovery cycles of human muscle action potentials and their sensitivity to ischemia. Muscle Nerve 2009;39:616–626. 19229874 10.1002/mus.21192

[23] Khogali S , Lucas B , Ammar T , Dejong D , Barbalinardo M , Hayward LJ , et al. Physiological basis for muscle stiffness and weakness in a knock‐in M1592V mouse model of hyperkalemic periodic paralysis. Physiol Rep 2015;3:e12656. 26702073 10.14814/phy2.12656 PMC4760441

[24] Lucas B , Ammar T , Khogali S , DeJong D , Barbalinardo M , Nishi C , et al. Contractile abnormalities of mouse muscles expressing hyperkalemic periodic paralysis mutant NaV1.4 channels do not correlate with Na+ influx or channel content. Physiol Genomics 2014;46:385–397. 24714718 10.1152/physiolgenomics.00166.2013

[25] Ingalls CP , Warren GL , Williams JH , Ward CW , Armstrong RB . E‐C coupling failure in mouse EDL muscle after in vivo eccentric contractions. J Appl Physiol Bethesda Md 1985 1998;85:58–67. 10.1152/jappl.1998.85.1.58 9655756

[26] Muscle Biopsy ‐ 5th Edition. https://www.elsevier.com/books/muscle‐biopsy/dubowitz/978‐0‐7020‐7471‐4.

[27] Phadke R . Myopathology of congenital myopathies: bridging the old and the new. Semin Pediatr Neurol 2019;29:55–70. 31060726 10.1016/j.spen.2019.01.007

[28] Bhatt DP , Chen X , Geiger JD , Rosenberger TA . A sensitive HPLC‐based method to quantify adenine nucleotides in primary astrocyte cell cultures. J Chromatogr B Analyt Technol Biomed Life Sci 2012;889–890:110–115. 10.1016/j.jchromb.2012.02.005 PMC3299834 22382093

[29] Boërio D , Corrêa TD , Jakob SM , Ackermann KA , Bostock H , Z'Graggen WJ . Muscle membrane properties in a pig sepsis model: effect of norepinephrine. Muscle Nerve 2018;57:808–813. 29130505 10.1002/mus.26013

[30] Sejersted OM , Sjøgaard G . Dynamics and consequences of potassium shifts in skeletal muscle and heart during exercise. Physiol Rev 2000;80:1411–1481. 11015618 10.1152/physrev.2000.80.4.1411

[31] Lamboley CR , Wyckelsma VL , Dutka TL , McKenna MJ , Murphy RM , Lamb GD . Contractile properties and sarcoplasmic reticulum calcium content in type I and type II skeletal muscle fibres in active aged humans. J Physiol 2015;11:2499–2514. 10.1113/JP270179 PMC4461411 25809942

[32] Lamboley CR , Wyckelsma VL , McKenna MJ , Murphy RM , Lamb GD . Ca leakage out of the sarcoplasmic reticulum is increased in type I skeletal muscle fibres in aged humans. J Physiol 2016;594:469–481. 26574292 10.1113/JP271382 PMC4713735

[33] Renganathan M , Messi ML , Delbono O . Dihydropyridine receptor‐ryanodine receptor uncoupling in aged skeletal muscle. J Membr Biol 1997;157:247–253. 9178612 10.1007/s002329900233

[34] Hanson MG , Wilde JJ , Moreno RL , Minic AD , Niswander L . Potassium dependent rescue of a myopathy with core‐like structures in mouse. Elife 2015;4:e02923. 10.7554/eLife.02923 PMC4309926 25564733

[35] Umanskaya A , Santulli G , Xie W , Andersson DC , Reiken SR , Marks AR . Genetically enhancing mitochondrial antioxidant activity improves muscle function in aging. Proc Natl Acad Sci 2014;111:15250–15255. 25288763 10.1073/pnas.1412754111 PMC4210348

[36] White Z , Terrill J , White RB , McMahon C , Sheard P , Grounds MD , et al. Voluntary resistance wheel exercise from mid‐life prevents sarcopenia and increases markers of mitochondrial function and autophagy in muscles of old male and female C57BL/6J mice. Skelet Muscle 2017;7:4. 28202058 10.1186/s13395-017-0120-3 PMC5311842

[37] The Jackson Laboratory . Aged C57Bl/6J mice for research studies. https://jackson.jax.org/rs/444‐BUH‐304/images/Whitepaper_Aged_B6.pdf.

[38] Lehmann‐Horn F , Küther G , Ricker K , Grafe P , Ballanyi K , Rüdel R . Adynamia episodica hereditaria with myotonia: a non‐inactivating sodium current and the effect of extracellular pH. Muscle Nerve 1987;10:363–374. 3587272 10.1002/mus.880100414

[39] Wu F , Mi W , Cannon SC . Bumetanide prevents transient decreases in muscle force in murine hypokalemic periodic paralysis. Neurology 2013;80:1110–1116. 23427324 10.1212/WNL.0b013e3182886a0e PMC3662304

[40] Wu F , Mi W , Cannon SC . Beneficial effects of bumetanide in a CaV1.1‐R528H mouse model of hypokalaemic periodic paralysis. Brain 2013;136:3766–3774. 24142145 10.1093/brain/awt280 PMC3859222

[41] Troni W , Doriguzzi C , Mongini T . Interictal conduction slowing in muscle fibers in hypokalemic periodic paralysis. Neurology 1983;33:1522–1525. 6685247 10.1212/wnl.33.11.1522

[42] Engel AG , Lambert EH . Calcium activation of electrically inexcitable muscle fibers in primary hypokalemic periodic paralysis. Neurology 1969;19:851–858. 5816878 10.1212/wnl.19.9.851

[43] Kleeman FJ , Partridge LD , Glaser GH . Resting potential and distribution of muscle fibers in living mammalian muscle. Am J Phys Med 1961;40:183–191. 14456709

[44] Andersson DC , Betzenhauser MJ , Reiken S , Meli AC , Umanskaya A , Xie W , et al. Ryanodine receptor oxidation causes intracellular calcium leak and muscle weakness in aging. Cell Metab 2011;14:196–207. 21803290 10.1016/j.cmet.2011.05.014 PMC3690519

[45] Shavlakadze T , Morris M , Fang J , Wang SX , Zhu J , Zhou W , et al. Age‐related gene expression signature in rats demonstrate early, late, and linear transcriptional changes from multiple tissues. Cell Rep 2019;28:3263–3273.e3. 31533046 10.1016/j.celrep.2019.08.043

[46] Jouaville LS , Pinton P , Bastianutto C , Rutter GA , Rizzuto R . Regulation of mitochondrial ATP synthesis by calcium: evidence for a long‐term metabolic priming. Proc Natl Acad Sci 1999;96:13807–13812. 10570154 10.1073/pnas.96.24.13807 PMC24146

[47] Matthews E , Neuwirth C , Jaffer F , Scalco RS , Fialho D , Parton M , et al. Atypical periodic paralysis and myalgia: a novel RYR1 phenotype. Neurology 2018;90:e412–e418. 29298851 10.1212/WNL.0000000000004894 PMC5791790

[48] Auré K , Dubourg O , Jardel C , Clarysse L , Sternberg D , Fournier E , et al. Episodic weakness due to mitochondrial DNA MT‐ATP6/8 mutations. Neurology 2013;81:1810–1818. 24153443 10.1212/01.wnl.0000436067.43384.0b

[49] Da Y , Lei L , Jurkat‐Rott K , Lehmann‐Horn F . Successful treatment of periodic paralysis with coenzyme Q10: two case reports. Acta Myol 2016;35:107–108. 28344441 PMC5343741

[50] Ke Q , Luo B , Qi M , Du Y , Wu W . Gender differences in penetrance and phenotype in hypokalemic periodic paralysis. Muscle Nerve 2013;47:41–45. 23019082 10.1002/mus.23460

[51] Barnes P . Skeletal muscle metabolism in myotonic dystrophy A 31P magnetic resonance spectroscopy study. Brain 1997;120:1699–1711. 9365364 10.1093/brain/120.10.1699

[52] Chinnery PF , Walls TJ , Hanna MG , Bates D , Fawcett P . Normokalemic periodic paralysis revisited: does it exist? Ann Neurol 2002;52:251–252. 12210802 10.1002/ana.10257

[53] Shi J , Qu Q , Liu H , Cui W , Zhang Y , Lv H , et al. SCN4A p.R675Q mutation leading to normokalemic periodic paralysis: a family report and literature review. Front Neurol 2019;10:1138. 31708864 10.3389/fneur.2019.01138 PMC6824318

[54] Lee YH , Lee H‐S , Lee HE , Hahn S , Nam T‐S , Shin HY , et al. Whole‐body muscle MRI in patients with hyperkalemic periodic paralysis carrying the *SCN4A* mutation T704M: evidence for chronic progressive myopathy with selective muscle involvement. J Clin Neurol 2015;11:331–338. 26256659 10.3988/jcn.2015.11.4.331 PMC4596100

[55] Von Haehling S , Morley JE , Coats AJS , Anker SD . Ethical guidelines for publishing in the Journal of Cachexia, Sarcopenia and Muscle: update 2017. J Cachexia Sarcopenia Muscle. 2017;8:1081–1083. 29098794 10.1002/jcsm.12261 PMC5700441

