# Supplementary figures and images for: Ageing contributes to phenotype transition in a mouse model of periodic paralysis
Source: JCSM Rapid Commun. 2021 May 5;4(2):245–59. doi: 10.1002/rco2.41 (PMC8837191; doi:10.1002/rco2.41)

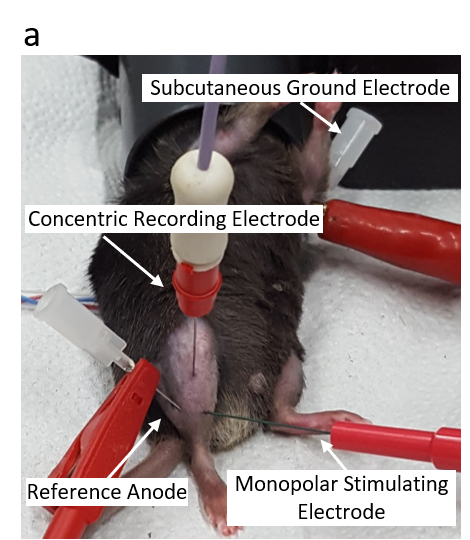

Supplement: Supplementary file 1 — Figure S1. Experimental setup for MVRCs in mouse muscle. a. Experimental set up for TA b. Experimental set up for triceps. In both A and B a monopolar stimulating needle electrode (28G TECA, Viasys Healthcare Madison, Wisconsin) was inserted into the distal muscle. A reference anode was inserted slightly above and lateral to the monopolar stimulating electrode. The reference anode consisted of a 27G hollow bore disposable steel needle attached to reference anode lead with crocodile clip. Stimuli consisting of 0.05 ms rectangular current pulses were delivered. Muscle activity was recorded with a concentric needle electrode (disposable 30G concentric EMG needle, TECA) inserted into the proximal end of the muscle. A ground electrode was inserted under the skin in the axilla. The ground electrode consisted of a 27G hollow bore disposable steel needle that was bent to make it easier to insert under the skin and attached to crocodile clip on the ground cable. [file RCO2-4-245-s003.zip › RCO2_41_Supp Fig 1a.tif]

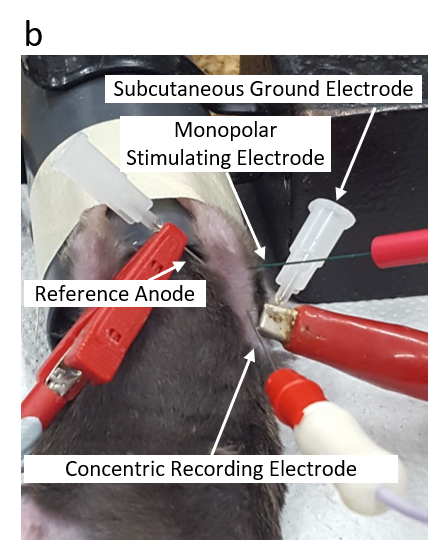

Supplement: Supplementary file 1 — Figure S1. Experimental setup for MVRCs in mouse muscle. a. Experimental set up for TA b. Experimental set up for triceps. In both A and B a monopolar stimulating needle electrode (28G TECA, Viasys Healthcare Madison, Wisconsin) was inserted into the distal muscle. A reference anode was inserted slightly above and lateral to the monopolar stimulating electrode. The reference anode consisted of a 27G hollow bore disposable steel needle attached to reference anode lead with crocodile clip. Stimuli consisting of 0.05 ms rectangular current pulses were delivered. Muscle activity was recorded with a concentric needle electrode (disposable 30G concentric EMG needle, TECA) inserted into the proximal end of the muscle. A ground electrode was inserted under the skin in the axilla. The ground electrode consisted of a 27G hollow bore disposable steel needle that was bent to make it easier to insert under the skin and attached to crocodile clip on the ground cable. [file RCO2-4-245-s003.zip › RCO2_41_Supp Fig 1b.tif]

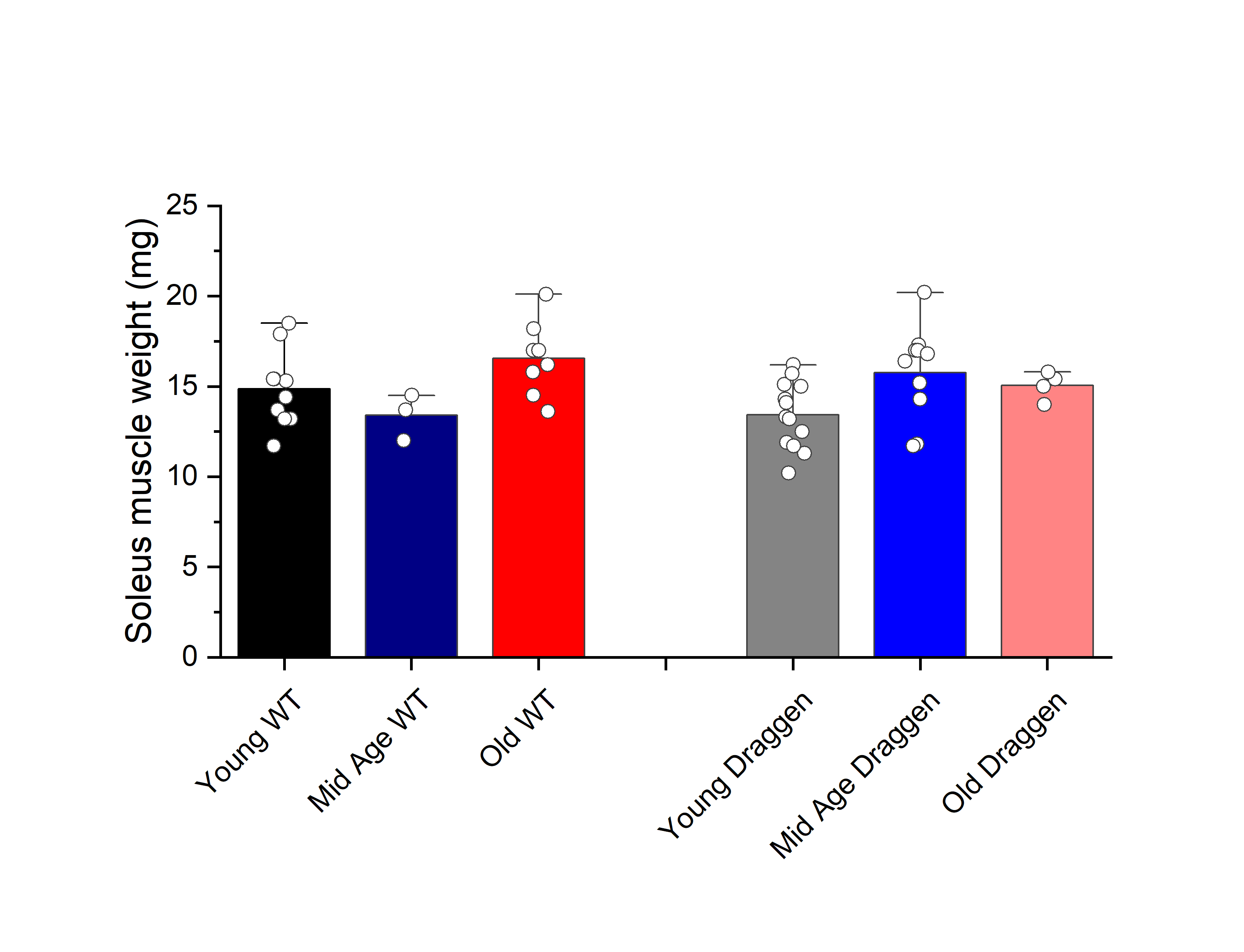

Supplement: Supplementary file 2 — Figure S2. Weight of dissected soleus muscle at time of terminal experiment in mice without access to a voluntary running wheel. [file RCO2-4-245-s001.png]
